# Tunable Luminescence in Sr_2_MgSi_2_O_7_:Tb^3+^, Eu^3+^Phosphors Based on Energy Transfer

**DOI:** 10.3390/ma10030227

**Published:** 2017-02-24

**Authors:** Minhong Li, Lili Wang, Weiguang Ran, Zhihan Deng, Jinsheng Shi, Chunyan Ren

**Affiliations:** Department of Chemistry and Pharmaceutical Science, Qindao Agricultural University, Qingdao 266109, China; 18765905832@163.com (M.L.); qauwanglili@126.com (L.W.); weiguangranqn@163.com (W.R.); ufodzh@sina.com (Z.D.)

**Keywords:** energy transfer, single-component, white light

## Abstract

A series of Tb^3+^, Eu^3+^-doped Sr_2_MgSi_2_O_7_ (SMSO) phosphors were synthesized by high temperature solid-state reaction. X-ray diffraction (XRD) patterns, Rietveld refinement, photoluminescence spectra (PL), and luminescence decay curves were utilized to characterize each sample’s properties. Intense green emission due to Tb^3+^ ^5^D_4_→^7^F_5_ transition was observed in the Tb^3+^ single-doped SMSO sample, and the corresponding concentration quenching mechanism was demonstrated to be a diople-diople interaction. A wide overlap between Tb^3+^ emission and Eu^3+^ excitationspectraresults in energy transfer from Tb^3+^ to Eu^3+^. This has been demonstrated by the emission spectra and decay curves of Tb^3+^ in SMSO:Tb^3+^, Eu^3+^ phosphors. Energy transfer mechanism was determined to be a quadrupole-quadrupole interaction. And critical distance of energy transfer from Tb^3+^ to Eu^3+^ ions is calculated to be 6.7 Å on the basis of concentration quenching method. Moreover, white light emission was generated via adjusting concentration ratio of Tb^3+^ and Eu^3+^ in SMSO:Tb^3+^, Eu^3+^ phosphors. All the results indicate that SMSO:Tb^3+^, Eu^3+^ is a promising single-component white light emitting phosphor.

## 1. Introduction

With the increasing seriousness of environmental problems and energy issues, white light emitting diodes (w-LEDs) have attract great attention in the lighting and display field due to their environmental friendliness, lower energy consumption, long lifetime, and extraordinary luminous efficiency compared with traditional incandescent or fluorescent lamps [1,2,3,4]. In general, three effective strategies can be used to generate white light. First is the combination of multiple LED chips (red, green, and blue) in a single device, called RGB-LEDs [5]. However, it is uneconomic to combine three or more LED chips to fabricate w-LEDs due to low efficiencies and expensive cost. The second approach to generate white light is the assembly of a single LED chip with red, green, and blue phosphors or a single-phase phosphor, which is called phosphor converted white LEDs (pc-WLEDs) [6]. Nowadays, leading commercial w-LEDs are fabricated by a “blue (InGaN) LED chip + yellow (YAG:Ce^3+^) phosphor” [7,8]. However, inherent weaknesses such as high correlated color temperature (CCT > 7000 K) and poor color rendering index (CRI < 80) were caused by the absence of red component, which greatly limiting its application [9,10]. In order to overcome these drawbacks, UV LED chip excited tricolor phosphors were prepared, which can provide high color-rendering index and quality of light [11]. However, poor luminescence efficiency was caused by mixing of multiemission bands, which contributed to strong reabsorption. As an alternative, it is obligatory to develop single-phase phosphor.

Rare earth ions doped silicate phosphors have been investigated extensively due to their cheap raw materials and good chemical and physical stability, which originates from the strong and rigid frameworks with covalent Si–O bonds [12]. Recently, Zhou et al. reported a single-component MgY_2_Si_3_O_10_:Bi^3+^, Eu^3+^ phosphor that can give white light emission under excitation of UV light and provide potential application for white-LEDs [13]. Effective energy transfer was observed from Eu^2+^ to Mn^2+^ in Mg_2_Al_4_Si_5_O_18_:Eu^2+^, Mn^2+^ phosphor, which was researched by Chen et al. [14]. In 2014, Wang et al. reported the luminescence properties of Y_2_SiO_5_:Ce^3+^, Tb^3+^, Eu^3+^ phosphor, which gives white light emission via energy transfer from Ce^3+^ to Tb^3+^ to Eu^3+^ [15]. In addition to similar host materials, on the other hand, energy transfer process was observed in rare earth co-doped phosphors. It is well known that energy transfer from sensitizers to activators plays an important role in realizing tunable emission [16]. For the rare earth family, trivalent Tb^3+^ ions have been widely studied due to ^5^D_3_→^7^F_J_ transitions in blue region and ^5^D_4_→^7^F*_J_* transitions in green region (*J* = 6, 5, 4 and 3) based on different doping concentration [17,18]. It can effectively transfer its energy to activators to improve the luminescence intensity of co-activators [19]. Eu^3+^ is an effective red component due to its ^5^D_0_→^7^F_2_ electric dipole transition, which subsititute sites without symmetry center. In order to realize tunable emission color, Tb^3+^ and Eu^3+^ co-doped SMSO (Sr_2_MgSi_2_O_7_) phosphors were prepared in this experiment. 

In this work, new single-phased SMSO:Tb^3+^, Eu^3+^phosphors were synthesized via high temperature solid state reaction. Crystal structure, photoluminescence properties, Commission International De L’Eclairage (CIE) chromaticity coordinates, and luminescence lifetimes have been investigated in detail. Energy transfer from Tb^3+^ to Eu^3+^ in SMSO:Tb^3+^, Eu^3+^ was investigated, and the corresponding energy transfer mechanism was determined to be a quadrupole-quadrupole interaction. Moreover, tunable emission from blue to white, up to red-orange, was observed under excitation of UV light. All results show that SMSO:Tb^3+^, Eu^3+^ is a potential single-component phosphor.

## 2. Results

### 2.1.Phase and Structure Analysis

**Figure 1:**
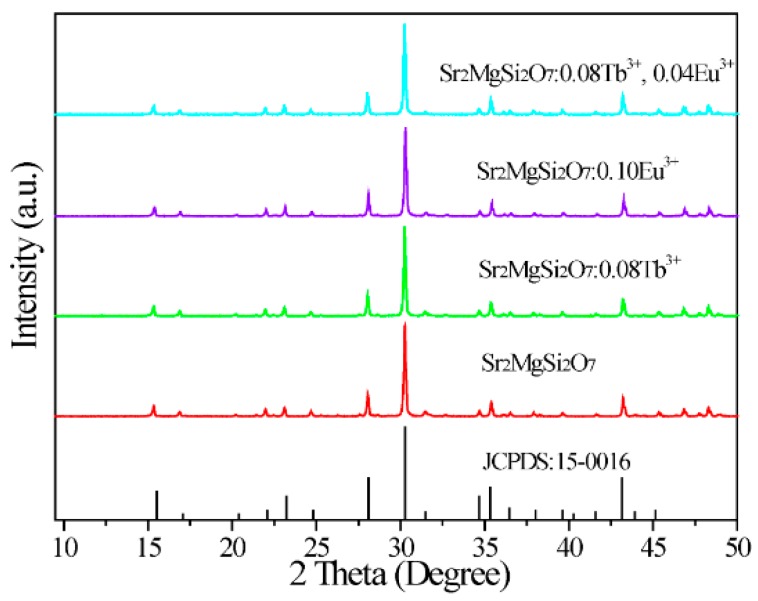
XRD patterns of SMSO (Sr_2_MgSi_2_O_7_) host, SMSO:0.08Tb^3+^, SMSO:0.10Eu^3+^ and SMSO:0.08Tb^3+^, 0.04Eu^3+^ phosphors.

Figure 1 gives the XRD patterns of Tb^3+^ and Eu^3+^ ions single-doped or co-doped SMSO phosphors prepared by high temperature solid state reaction at 1300 °C for 3 h. It can be found that all diffraction peaks of phosphors matched well with SMSO phase (JCPDS#15-0016), demonstrating that prepared samples are single-component and a small quantity of Tb^3+^ and Eu^3+^ ions will not induce any other significant changes for SMSO lattice.

**Figure 2:**
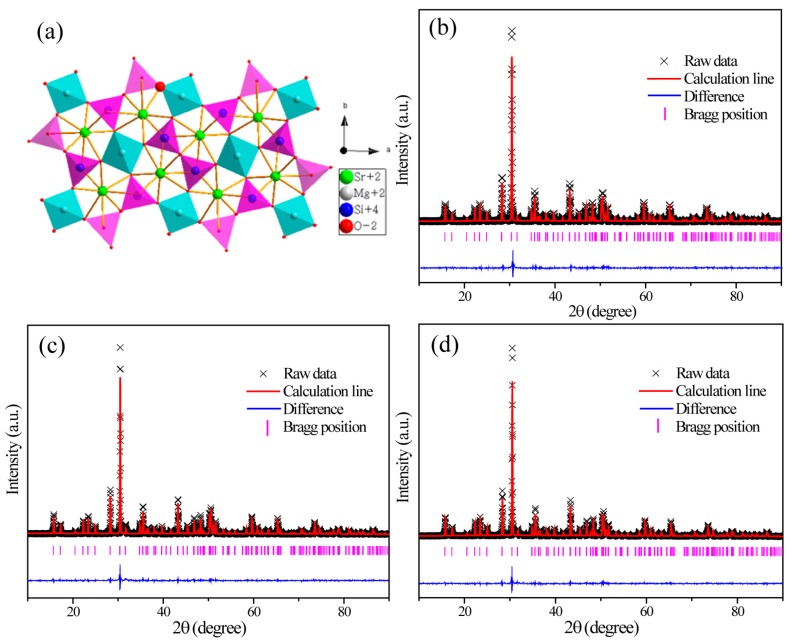
(**a**) Crystal structure of SMSO. Experimental (black crosses) and calculated (red solid line) XRD patterns and their difference (blue solid line) for (**b**) SMSO:0.08Tb^3+^; (**c**) SMSO:0.10Eu^3+^ and (**d**) SMSO:0,08Tb^3+^, 0.04 Eu^3+^ samples by the GSAS program. The short magenta vertical lines show the position of Bragg reflections of the calculated patterns.

Figure 2a depicts the crystal structure of SMSO crystallizes in a tetragon, with cell parameters of *a* = *b* = 8.01 Å, *c* = 5.16 Å, *V* = 331.34 Å^3^, *Z* = 2. Rare earth ions preferred to occupy Sr^2+^ rather than Mg^2+^ sites because of similar ionic radius of Sr^2+^ (*r* = 1.26 Å for *CN* = 8), Mg^2+^ (*r* = 0.57 Å for *CN* = 4), Tb^3+^ (*r* = 1.04 Å for *CN* = 8) and Eu^3+^ (*r* = 1.06 Å for *CN* = 8) [20]. In order to further identify the influence of doping ions on crystal structure, structure refinement of powder XRD patterns of SMSO:0.08Tb^3+^, SMSO:0.10Eu^3+^ and SMSO:0.08Tb^3+^, 0.04Eu^3+^ samples were performed by the general structure analysis system (GSAS) method. The final results were summarized in Table 1. The original structure model with crystallographic data of SMSO (ICSD #155330) was used to refine the above samples. Corresponding patterns for Rietveld refinements of SMSO:0.08Tb^3+^, SMSO:0.10Eu^3+^ and SMSO:0.08Tb^3+^, 0.04Eu^3+^ samples at room temperature are displayed in Figure 2b,c, respectively. The results indicate that rare earth ions doped SMSO phosphors with space group of P-421m have a tetragonal structure. 

### 2.2. Photoluminescence and Energy Transfer 

**Figure 3:**
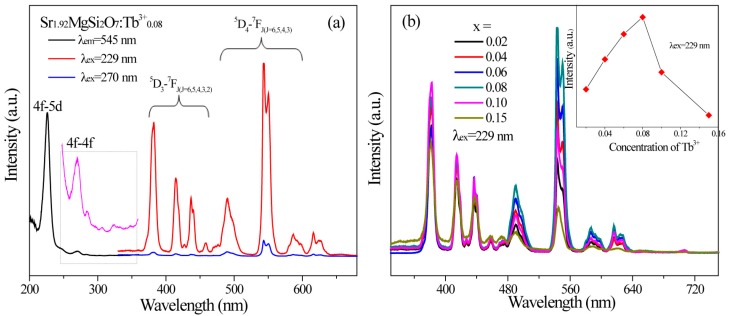
(**a**) The excitation and emission spectra of SMSO:0.08Tb^3+^ sample; (**b**) The emission spectra of SMSO:*x*Tb^3+^ (*x* = 0.02, 0.04, 0.06, 0.08, 0.10 and 0.15) samples.

Figure 3a gives the excitation and emission spectra of SMSO:0.08Tb^3+^ phosphor. The excitation spectrum monitored at 545 nm shows a broad band ranging from 200 to 250 nm with the maximum at 229 nm, which originated from 4f-5d spin-allowed transition of Tb^3+^. Other weak absorption bands in the region of 250 to 350 nm are ascribed to 4f-4f spin-forbidden transitions. When excited at 229 nm, the emission spectrum composes of both ^5^D_3_→^7^F*_J_* (*J* = 3, 4 and 5) and ^5^D_4_→^7^F*_J_* (*J* = 3, 4, 5 and 6) transitions. It can be observed that there is no other important change for Tb^3+^ emission except for the luminescence intensity under different excitation conditions. 

**Figure 4:**
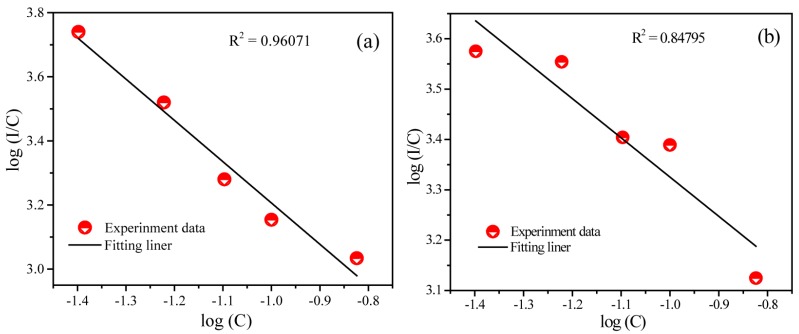
Dependence of lg(*I/C*) on lg(*C*) for SMSO:xTb^3+^ phosphors. ((**a**) represents concentration of blue emission; (**b**) represents concentration of green emission; C represents the concentration of Tb^3+^).

Figure 3b displays the emission spectra of SMSO:*x*Tb^3+^samples with different Tb^3+^ concentration under excitation of 229 nm. The inset shows the change of luminescence intensity of Tb^3+^ according to different Tb^3+^doping concentration. It can be seen that luminescence intensity of SMSO:*x*Tb^3+^ samples increases gradually with increasing Tb^3+^ content from 0 to 8 mol %, and then decreases when the concentration of Tb^3+^ is enhanced over 8 mol %. It is due to the concentration quenching effect, which is assigned to non-irradiative energy transfer between adjacent Tb^3+^ ions. For investigating the concentration quenching mechanism, it is necessary to calculate the critical distance (*R_c_*) between Tb^3+^ ions. According to concentration quenching theory, R_c_ was determined by [21,22]:

(1) $$Rc=2{\left[\frac{3V}{4\pi X_{c}N}\right]}^{1/3}$$

where *V* corresponds to the volume of unit cell, *N* is the number of host cations in the unit cell, and *X_c_* is the critical concentration of dopant ions. In this paper, *V* = 331.34 Å^3^, *N* = 2 and *X_c_* is 0.08 for Tb^3+^ doped SMSO phosphor, as a consequence, *R_c_* was calculated to be 7.9 Å. Generally, non-radiative energy transfer was occurred due to exchange interaction, radiation re-absorption, and electric multipolar interactions [23]. For SMSO:*x*Tb^3+^ samples, *R_c_* was calculated to be 7.9 Å. As a consequence, we can speculate that exchange interaction is weak in this sample since exchange interaction occurred when *R_c_* less than 5 Å [24]. Also, radiation re-absorption plays no role in Tb^3+^ concentration quenching process because of poor overlap between Tb^3+^ excitation and emission spectra. In consequence, electric multipolar interaction is major in Tb^3+^ concentration quenching. According to Dexter’s energy transfer theory, the concentration quenching mechanism for SMSO:*x*Tb^3+^ phosphors was calculated by the follow equation [25]:

*I*/*C* = *k*_1_/β × C*^s^*^/3^ (2)

where *I* is the emission intensity of activator, *C* is the related concentration of Tb^3+^, *k*_1_ and *β* are constants for each interaction under the same excitation wavelength in SMSO matrix, and s represents the different electric multipolar interactions which is that when *s* are equal to 6, 8 and 10, corresponding to dipole–diople (d–d), diople−quadrupole (d–q), and quadrupole–quadrupole (q–q) interacitions, respectively [26]. Figure 4 gives the linear relationship of log(*I*/*C*) versus log(*C*) in SMSO:Tb^3+^ phosphor. The value of s was calculated to be 5.66 (blue emission) and 5.31 (green emission), indicating that d-d interaction is major concentration quenching mechanism for SMSO:Tb^3+^ phosphor. 

**Figure 5:**
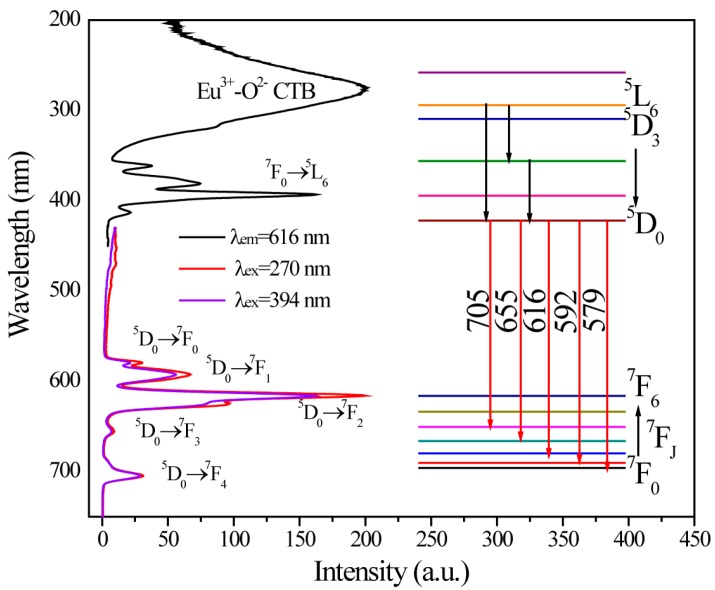
The excitation and emission spectra of SMSO:0.10Eu^3+^ sample and simple energy level transitions of Eu^3+^. (Note: The all energy levels were not positioned in an energy level diagram due to electrons transitions.)

Figure 5 exhibits the excitation and emission spectra of SMSO:0.10Eu^3+^ sample and simple energy level transitions of Eu^3+^, respectively. Broad excitation band from 200 to 450 nm was observed monitored at 616 nm. The strongest peak at about 270 nm is ascribed to Eu^3+^−O^2−^ charge transfer transition (CTB) from negative oxygen 2p orbit to the empty 4f orbit of Eu^3+^, which is easy to influence by host environment [27]. Other narrow absorption peaks in the regionof 300 to 450 nm at 363, 382, 394, and 467 nm are attributed to ^7^F_0_→^5^D_4_, ^7^F_0_→^5^G_4_, ^7^F_0_→^5^L_6_, and ^7^F_0_→^5^D_2_ transitions, respectively. Under excited at 270 nm, SMAO:0.10Eu^3+^ phosphor represents a series of narrow emission lines ranging from 500 to 750 nm at about 579, 592, 616, 654, and 705 nm, which corresponding to ^5^D_0_→^7^F*_J_* (*J* = 0, 1, 2, 3, and 4) transitions. And we can find that the position of emission peak don’t occur obvious migration under different excitation wavelength.

**Figure 6:**
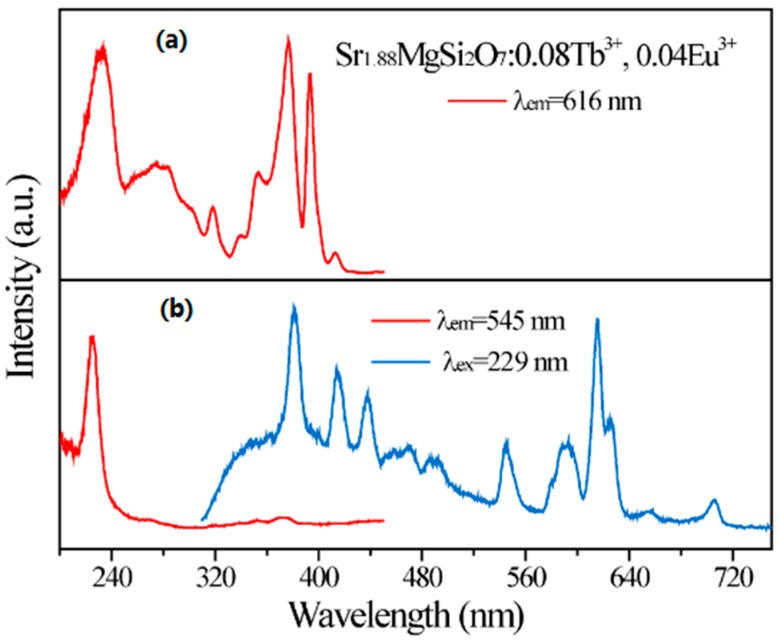
(**a**) The excitation spectrum of SMSO:Tb^3+^, Eu^3+^ phosphor monitored at 616 nm; (**b**) The excitation spectrum (red line) of SMSO:Tb^3+^, Eu^3+^ phosphor monitored at 545 nm and emission spectrum (blue line) of SMSO:Tb^3+^, Eu^3+^ phosphor excited at 229 nm.

The important spectra overlap of Tb^3+^ emission and Eu^3+^ excitation bands was observed from Figure 3b and Figure 5, indicating that there may exist energy transfer from Tb^3+^ to Eu^3+^. In consequence, Tb^3+^ was introduced into SMSO:Eu^3+^ phosphor to improve the red emission of Eu^3+^. Figure 6 gives the excitation and emission spectra of SMSO:0.08Tb^3+^, 0.04Eu^3+^ phosphor. Excited at 229 nm, as-prepared sample not only displays Tb^3+^ characteristic emissions in blue and green bands, but also gives a strong red emission band with the center at 616 nm from Eu^3+^. Monitoring at 545 nm emission from Tb^3+^, excitation spectrum shows similar profiles with Tb^3+^ single-doped SMSO sample. Monitored at 616 nm emission from Eu^3+^, the excitation spectrum consists of Tb^3+^ absorption peak at 229 nm, Eu^3+^−O^2−^ charge transfer band at 270 nm as well as other sharp emission lines from Eu^3+^, which gives direct evidence of energy transfer from Tb^3+^→Eu^3+^.

**Figure 7:**
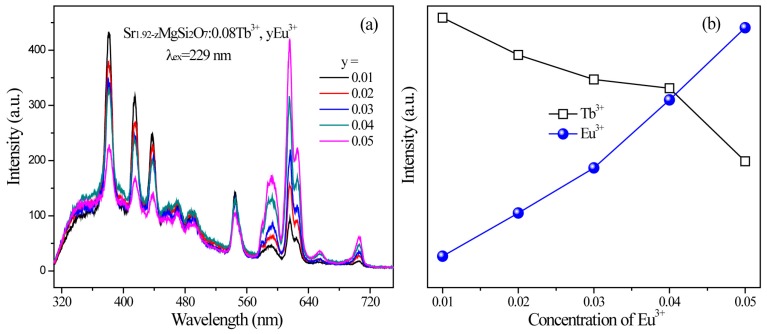
(**a**) The emission spectra of SMSO:0.08Tb^3+^, *y*Eu^3+^ (*y176815* = 0.01, 0.02, 0.03, 0.04 and 0.05) phosphors excited at 229 nm; (**b**) The changing of luminescence intensities of Tb^3+^ and Eu^3+^ based on different Eu^3+^ concentration.

The emission spectra of SMSO:0.08Tb^3+^, yEu^3+^ phosphors with fixed Tb^3+^ concentration and changed Eu^3+^ concentration were reveled in Figure 7a. We can observe that the emission intensities of Tb^3+^ decrease gradually while the luminescence intensities of Eu^3+^ increase monotonously with increasing Eu^3+^ doping content, which can be observed intuitively from Figure 7b. The result shows that energy transfer process occurs from Tb^3+^ to Eu^3+^ ions in SMSO:Tb^3+^, Eu^3+^ phosphors. The luminescence intensity of Eu^3+^ was improved 2 times compared with Eu^3+^ single-doped sample.

**Figure 8:**
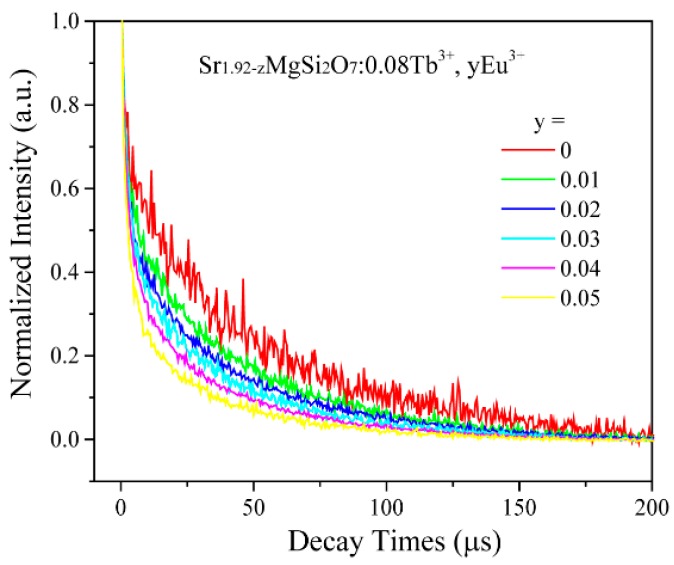
PL decay curves of Tb^3+^ in SMSO:0.08Tb^3+^, *y*Eu^3+^ samples under 229 nm radiations.

To further confirm energy transfer behavior from Tb^3+^ to Eu^3+^, luminescence lifetimes of Tb^3+^ ^5^D_4_→^7^F_5_ transition at 545 nm were measured in SMSO:0.08Tb^3+^, *y*Eu^3+^ samples under the excitation of 229 nm UV light. As demonstrated in Figure 8, the fluorescent decay curves are described distinctly with increasing Eu^3+^ doping concentration. Therefore, the luminescence lifetimes of Tb^3+^ revealed double-exponential types in all samples. The luminescence curves can be matched well with double-exponential expression:

*I*(*t*) = *I*_0_ + *A*_1_exp(−*t*/τ_1_) + *A*_2_exp(−*t*/τ_2_) (3)

where *I* and *I*_0_ represents the luminescence intensity at time *t* and 0, *A*_1_ and *A*_2_ are constants, *t* represents the time, and τ_1_ and τ_2_ represents the luminescence lifetimes for the exponential composition. As a function of these parameters, the average luminescence lifetimes (τ) was determined as follow equation:

τ = (*A*_1_τ_1_^2^ + *A*_2_τ_2_^2^)/(*A*_1_τ_1_ + *A*_2_τ_2_) (4)

For SMSO:0.08Tb^3+^, *y*Eu^3+^ phosphors, average lifetimes of Tb^3+^ emission under excitation of 229 nm were calculated to be 32.58, 24.48, 18.19, 12.09,9.21 and 7.59 µs when Eu^3+^ concentration were 0, 0.01, 0.02, 0.03, 0.04 and 0.05, respectively, indicating that energy transfer occurred from Tb^3+^ to Eu^3+^, as prospective.

**Figure 9:**
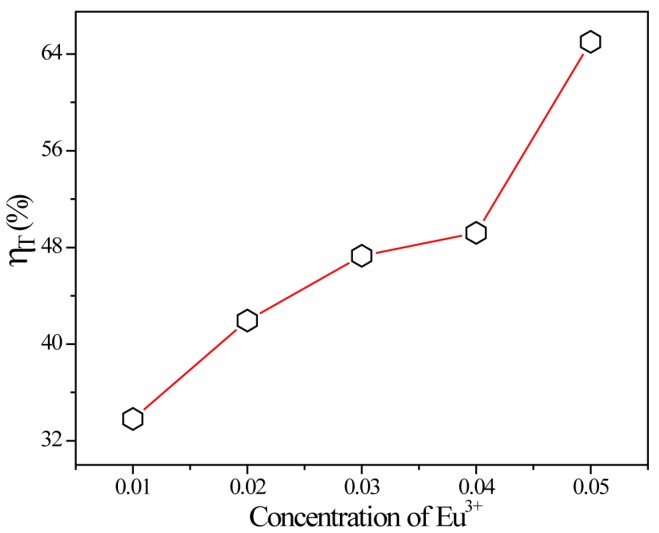
Energy transfer efficiencies (*η_T_*) from Tb^3+^ to Eu^3+^ in SMSO:0.08Tb^3+^, yEu^3+^ phosphors as a function of different Eu^3+^ concentration.

Moreover, the energy transfer efficiency (*η*) from Tb^3+^ to Eu^3+^ in SMSO:0.08Tb^3+^, *y*Eu^3+^ matrixwas calculated by the expression as follow [28]:

*η_ET_* = 1 − *I_S_*/*I_S_*_0_ (5)

where *η_ET_* represents the energy transfer efficiency, *I_S_* and *I_S_*_0_ represent the corresponding luminescence intensities of Tb^3+^ in the presence and absence of the Eu^3+^, respectively. Figure 9 reveals the energy transfer efficiency from Tb^3+^ to Eu^3+^ in SMSO:0.08Tb^3+^, yEu^3+^ samples excited at 229 nm based on different Eu^3+^ concentration. It can be seen that *η_ET_* increases monotonously within corporation Tb^3+^ into SMSO:Eu^3+^ sample, which can reach the maximum value of 65%. Therefore, the energy transfer from Tb^3+^ to Eu^3+^ ions is efficient to improve Eu^3+^ luminescence.

**Figure 10:**
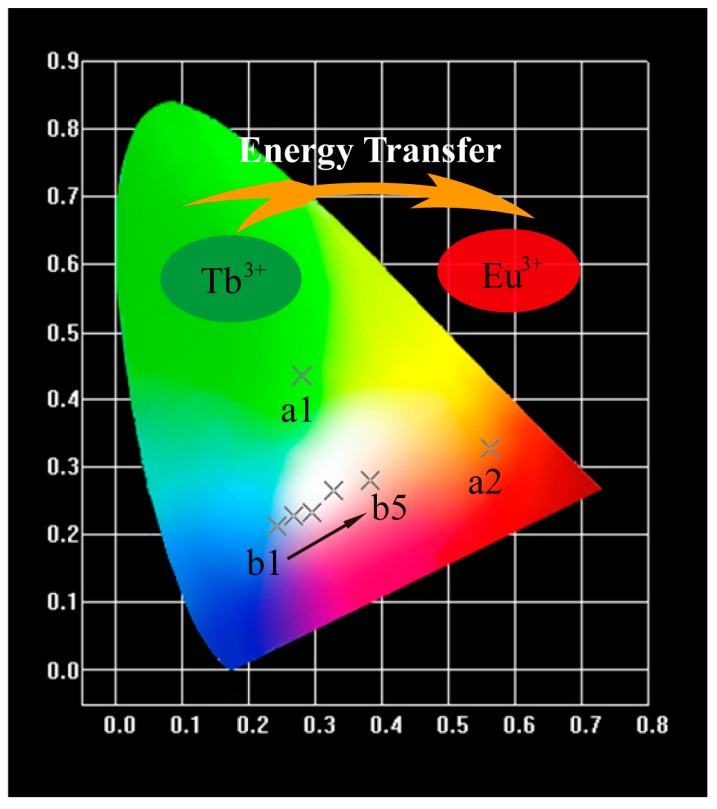
CIE chromaticity coordinates for SMSO:0.08Tb^3+^ (a1), SMSO:0.10Eu^3+^ (a2) and SMSO:0.08Tb^3+^, *y*Eu^3+^ (b1–b5) phosphors.

According to the Commission International De L’Eclairage 1931 chromaticity coordinates, the CIE chromaticity diagram of Tb^3+^ and Eu^3+^ single-doped or co-doped SMSO samples were portrayed in Figure 10 and corresponding values were summarized in Table 2. Under excitation of 229 nm, SMSO:0.08Tb^3+^ displays intense blue-green emission, while SMSO:0.10Eu^3+^ sample gives bright red emission excited at 270 nm. Furthermore, we can observe that the emission color was varied from blue to white, eventually to red-orange light with enhancing Eu^3+^ doping concentration from 0.01 to 0.05 in Tb^3+^ and Eu^3+^ co-doped phosphors. It is due to energy transfer from Tb^3+^ to Eu^3+^ ions. And CIE chromaticity coordinate of SMSO:0.08Tb^3+^, 0.04Eu^3+^ sample is close to standard white light.

**Figure 11:**
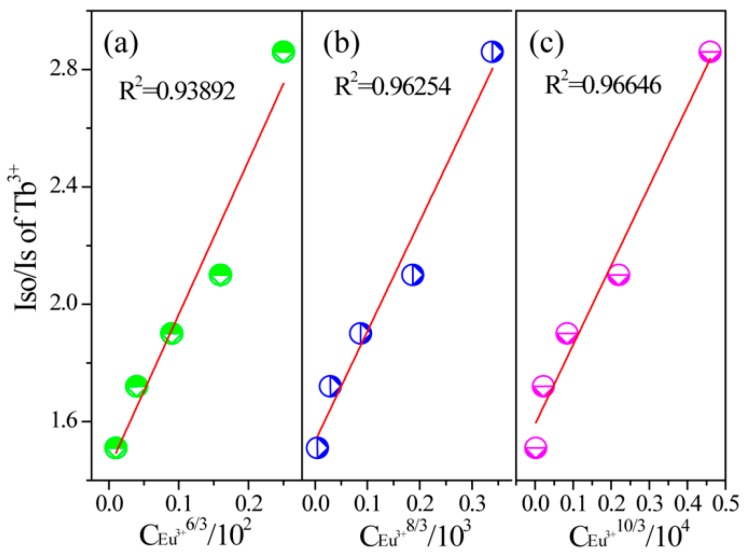
Dependence of *I_S_*_0_/*I_S_* of Tb^3+^ on (**a**) C_Eu3+_^6/3^; (**b**) C_Eu3+_^8/3^; and (**c**) C_Eu3+_^10/3^.

As revealed above, critical distance R_c_ was calculated to be 6.7 Å as a function of Equation (1) for SMSO:0.08Tb^3+^, *y*Eu^3+^ samples, in which the different *X_c_* was contributed to critical content (*X_c_* was total concentration of Tb^3+^ and Eu^3+^ ions, *X_c_* = 0.13). The result shows that electric multipole interaction plays an important role in energy transfer process from Tb^3+^ to Eu^3+^. On account of Dexter’s energy transfer mechanism for multipolar interaction and Reisfeld’s approximation, the following expression can be given:

*I_S_*_0_/*I_S_*∝*C*^θ/3^ (6)

where *I_S_*_0_ and *I_S_* are the luminescence intensity of sensitizer without and with activator. *C* represents the doping concentration of Tb^3+^ and Eu^3+^ ion. The value for θ = 6, 8 and 10 corresponding to dipole-dipole, dipole-quadrupole, and quadrupole-quadrupole interactions. The linear relation of *I_S_*_0_/*I_S_* and *C*^θ/3^ are revealed in Figure 11. R^2^ value was calculated to be 0.96646 when θ = 10 for SMSO:0.08Tb^3+^, *y*Eu^3+^ samples, demonstrating that the energy transfer from Tb^3+^→Eu^3+^ was evaluated to be a quadrupole-quadrupole interaction.

## 3. Materials and Methods 

### 3.1. Sample Preparation

A series of Sr_2_MgSi_2_O_7_:Tb^3+^, Eu^3+^ phosphors were prepared by high temperature solid state reaction in air atmosphere at 1300 °C. SrCO_3_ (A.R.), MgO (A.R.), SiO_2_ (A.R.), Tb_4_O_7_ (99.99%), and Eu_2_O_3_ (99.99%) were used as raw materials. They were weighed according to desired composition and mixed thoroughly in ball mill with appropriate ethanol for 4 h, then the powder samples were moved to culture dish to dry for 1.0 h at 60 °C. After that, they were transferred into ceramic crucible and calcined in high temperature tubular furnace at 1300 °C for 3 h. The final samples were obtained by regrinding for 3 min.

### 3.2. Measurements and Characterization

Bruker D8 Focus diffractmeter (voltage 40 kV and current 40 mA) at a scanning rate of 10 deg/min over the 2θ range from 10° to 50° with graphite monochromatized CuKα radiation (λ = 0.15405 nm) was used to record XRD patterns of SMSO:Tb^3+^, Eu^3+^ samples. F-4600 device (FL-Spectorphotomet) with a 150 W xenon lamp light source was used to measure the excitation and emission spectra. Structure refinement of SMSO, SMSO:Tb^3+^, SMSO:Eu^3+^ and SMSO:Tb^3+^, Eu^3+^sampleswere performed by GSAS (General Structure Analysis System) program with radiation at a 0.01°(2θ)/0.1 s scanning step. UV-vis diffuse reflectance spectra were measured using a UV–Vis spectrophotometer (TU–1901).

## 4. Conclusions

In summary, a series of Tb^3+^ and Eu^3+^ doped SMSO phosphors were prepares by high temperature solid-state reaction at 1300°C for 3 h. The characteristic emissions of Tb^3+^ (blue, ^5^D_3_→^7^F_3_ and green, ^5^D_4_→^7^F_5_) and Eu^3+^ (red, ^5^D_0_→^7^F_2_) were observed in SMSO:Tb^3+^ and SMSO:Eu^3+^ samples, respectively. For the SMSO:Tb^3+^, Eu^3+^ sample, efficient energy transfer was observed from Tb^3+^ to Eu^3+^, which is deduced by the spectra overlap of Tb^3+^ emission and Eu^3+^ excitation. This was further proved by the emission spectra and decay curves of Tb^3+^ in the SMSO:Tb^3+^, Eu^3+^ sample. The corresponding energy transfer mechanism was demonstrated to be a quadrupole-quadrupole interaction. The emission color was tuned from green to white, up to the red region, by adjusting the concentration ratio of Tb^3+^ and Eu^3+^ in SMSO:Tb^3+^, Eu^3+^ phosphors. All results indicate that SMSO:Tb^3+^, Eu^3+^ is a promising single-component white light emission phosphor.

## Figures and Tables

**Table 1:** Refinement, Crystallographic, and Structure. Parameters of the SMSO:0.08Tb^3+^, SMSO:0.10Eu^3+^, and SMSO:0.08Tb^3+^, 0.04Eu^3+^ samples.

Formula	SMSO	SMSO:0.08Tb	SMSO:0.10Eu	SMSO:0.08Tb, 0.04Eu
Crystal System	Tetragonal	Tetragonal	Tetragonal	Tetragonal
Space Group	P-421m (113)	P-421m (113)	P-421m (113)	P-421m (113)
*a*/Å	8.01	8.0111	8.0112	8.0108
*b*/Å	8.01	8.0111	8.0112	8.0108
*c*/Å	5.16	5.1667	5.1657	5.1650
*v*/Å^3^	331.34	331.58	331.53	331.45
*Z*	2	2	2	2
Radiation Type	-	Cu−Kα	Cu−Kα	Cu−Kα
Wavelength/Å	-	1.5405	1.5405	1.5405
Profile Range/°	-	10°−90°	10°−90°	10°−90°
Rp/%	-	8.75	8.46	7.97
Rwp/%	-	11.95	11.51	10.9
χ^2^	-	3.552	3.721	4.157

**Table 2:** CIE chromaticity coordinates and color temperature for SMSO:0.08Tb^3+^, *y*Eu^3+^ phosphors excited at 229 nm.

Sample No.	SMSO:*x*Tb^3+^, *y*Eu^3+^	CIE (*x*, *y*)	CCT (K)
a1	*x* = 0.08, *y* = 0	(0.2796, 0.4359)	7191
a2	*x* = 0, *y* = 0.10	(0.5646, 0.3272)	4937
b1	*x* = 0.08, *y* = 0.01	(0.2424, 0.2137)	164,473
b2	*x* = 0.08, *y* = 0.02	(0.2682, 0.2263)	31,313
b3	*x* = 0.08, *y* = 0.03	(0.2943, 0.2337)	13,267
b4	*x* = 0.08, *y* = 0.04	(0.3271, 0.2644)	5827
b5	*x* = 0.08, *y* = 0.05	(0.3821, 0.2857)	2768
